# The Effectiveness of Denosumab in Middle Eastern Patients With Giant Cell Tumor of the Bone: A Single-Center, Retrospective Study

**DOI:** 10.7759/cureus.58292

**Published:** 2024-04-15

**Authors:** Mahmoud Elshenawy, Tarek Z Arabi, Heba A Ateya, Tusneem Elhassan, Saad S Ali, Rana K Othman, Radwan Alkhatib, Ayman Elshentenawy, Ahmed Badran

**Affiliations:** 1 Department of Medical Oncology, King Faisal Specialist Hospital and Research Centre, Riyadh, SAU; 2 Department of Clinical Oncology and Nuclear Medicine, Menoufiya University, Shebin El Kom, EGY; 3 College of Medicine, Alfaisal University, Riyadh, SAU; 4 Department of Medical Oncology, Cairo University, Cairo, EGY; 5 Department of Clinical Oncology and Nuclear Medicine, Faculty of Medicine, Ain Shams University, Cairo, EGY

**Keywords:** saudi arabia, middle east, progression-free survival, denosumab, giant cell tumor of the bone

## Abstract

Background: Giant cell tumor of the bone (GCTB) is an aggressive benign tumor, which constitutes 5% of all primary bone tumors. Denosumab, a receptor activator of nuclear factor κB ligand monoclonal antibody, inhibits osteoclast-induced bone destruction and has demonstrated promising results in patients with GCTB. However, the long-term efficacy of the drug has not been extensively studied, especially in the Middle East.

Methodology: In this study, we retrospectively analyzed the five-year progression-free survival (PFS) in patients with GCTB at a single Saudi center. PFS was defined as the time from diagnosis until disease progression, relapse, or death. Events were censored after five years from diagnosis.

Results: Sixty-two patients with GCTB were included in the study. The median age at diagnosis was 31.16 years, and 38 (61.3%) patients were female. Twenty-nine patients (46.8%) received denosumab during the study period. The median duration of denosumab treatment was 5.06 months, and the median number of cycles was 6. The median PFS was not reached, and the five-year PFS rate was 60.3%. Age, gender, body mass index, performance status at presentation, and tumor location had no impact on five-year PFS. Denosumab treatment prolonged PFS; however, this was not statistically significant compared to non-denosumab patients (*P *= 0.603).

Conclusions: Denosumab does not seem to provide superior long-term outcomes compared to surgery alone. Although our findings are generally consistent with other studies in the literature, larger long-term studies are needed to confirm our findings.

## Introduction

Giant cell tumors of the bone (GCTB) are aggressive benign tumors constituting 5% of primary bone tumors and 20% of all benign bone neoplasms with recurrence rates reaching 60% [1,2]. GCTB most commonly arises near the knee in the second and third decades of life [2]. Histologically, GCTB consists of multinucleated osteoclast-like giant cells and mononuclear stromal cells [2]. Surgical resection is the first-line treatment for GCTB; however, some patients present with unresectable or metastatic diseases. Therefore, recent therapies have aimed to reduce neoplasm size before surgery. Denosumab, a receptor activator of nuclear factor κB ligand (RANKL) monoclonal antibody, inhibits osteoclastic activity and osteoclast-induced bone destruction [3]. Denosumab has been approved by the United States Food and Drug Administration for the treatment of osteoporosis in postmenopausal women and to increase bone mass in breast and prostate cancer patients [4]. Recently, the United States Food and Drug Administration approved the use of denosumab in locally advanced or metastatic GCTB [3]. Denosumab can also be used to reduce blood supply to neoplasms, allowing for easier tumor resection [5]. Although recent studies have shown promising outcomes with denosumab, the long-term effects of denosumab have not been studied extensively. Additionally, studies analyzing the long-term effects of denosumab in the Middle Eastern region are limited by their small sample size [6]. Therefore, in this study, we compare the progression-free survival (PFS) of patients with GCTB who received denosumab versus those who did not.

## Materials and methods

We retrospectively reviewed the medical files of all patients with GCTB at our center between May 2008 and December 2022. All patients with histological confirmation of GCTB at our center were included in the study. We included some patients from a previous study that studied the prognosis of patients with GCTB on denosumab [6]. One of the limitations of the previous study is the smaller proportion of denosumab patients compared to non-denosumab patients. Hence, we aimed to include a more balanced representation of denosumab patients in this study.

Approval for this study was received by our Institutional Review Board (IRB) at King Faisal Specialist Hospital and Research Center, Riyadh (#2161166). Patient consent was waived due to the retrospective nature of the study. Denosumab has been authorized in our hospital as a standard of care for the treatment of patients diagnosed with advanced GCTB at our center. It is administered 120 mg subcutaneously once every 28 days, with two additional loading doses given on days 8 and 15 of the first month. Patients were followed up every two weeks for the first month, and every month after that. Routine laboratory and physical examinations were conducted at each visit. An X-ray scan was done every three months of follow-up.

Patient demographics, including age, gender, and body mass index, were collected. Performance status (PS) at presentation was assessed according to the Eastern Cooperative Oncology Group scale [7]. Response to treatment was evaluated using Response Evaluation Criteria in Solid Tumors version 1.1 [8]. PFS was defined as the duration from the start of treatment until tumor progression, recurrence, or death. Furthermore, we collected data regarding the disease course, such as the tumor size, relapse, and therapeutic modalities.

Statistical analysis

Statistical analyses were conducted using SPSS version 26 (IBM Corp., Armonk, NY). Continuous variables were reported as a median. We applied the Kaplan-Meyer method for survival curves and tables, and the log-rank test was used to compare the various subgroups in our study. Events were censored after five years from diagnosis. A univariate Cox proportional hazards model was also utilized to compare the effect of denosumab in these patients. The *P*-value threshold for statistical significance was established at <0.05.

## Results

Sixty-two patients with GCTB were included in the study. The median age at diagnosis was 31.16 years. The median follow-up period was 6.3 years. In our study, 38 patients (61.3%) were female, 35 (56.5%) had a normal body mass index (BMI), and 52 (83.9%) of the subjects had a PS of 1 at presentation. Thirty-one (50%) had the tumor in the upper limb, and 26 (41.9%) in the lower limb. The median size of the tumors was 7 cm (range 1.5-16 cm).

**Figure 1:**
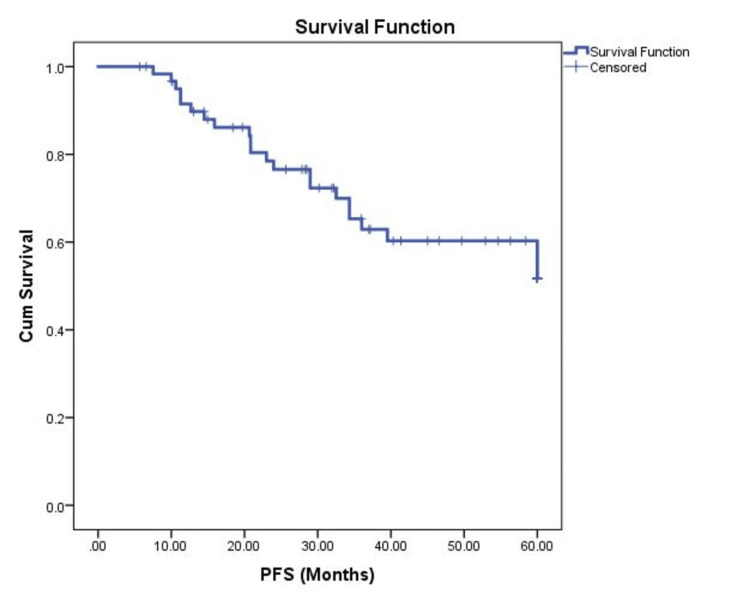
Kaplan-Meier analysis showing PFS of the patient population. PFS, progression-free survival; Cum, cumulative

Twenty-nine patients (46.8%) received denosumab during the study period. The median duration of denosumab treatment was 5.06 months, and the median number of cycles was 6 (range 2-25 cycles). Fifty-eight patients (94%) underwent surgery. Only 8 (12.9%) patients had post-surgical residual tumors. Twenty-two patients (35.5%) had post-primary relapse, of which 12 (54.4%) had localized relapse. Half of the relapse patients underwent surgery post-relapse. Similarly, half of the relapse patients received post-relapse denosumab, with a median treatment duration of 2.76 months (range 1.84-20.24). The median number of post-relapse denosumab cycles was 5 (range 4-18). The median PFS was not reached, and the five-year PFS rate was 60.3% (Figure 1).

**Figure 2:**
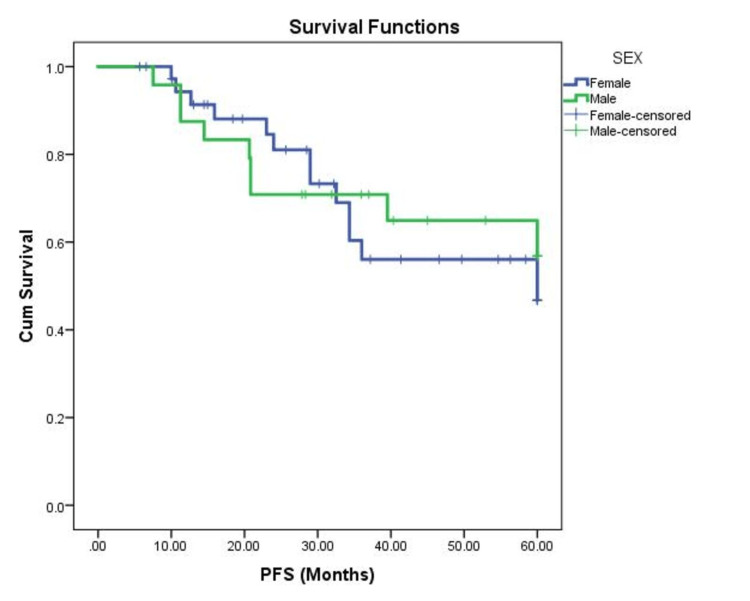
Kaplan-Meier analysis comparing the PFS of male and female patients with GCTB. PFS, progression-free survival; Cum, cumulative; GCTB, giant cell tumors of the bone

There was no significant difference in PFS between the two sexes (*P *= 0.75) (Figure 2) and different age groups (*P *= 0.178). The patient BMI category was not associated with changes in PFS (*P *= 0.534).

**Figure 3:**
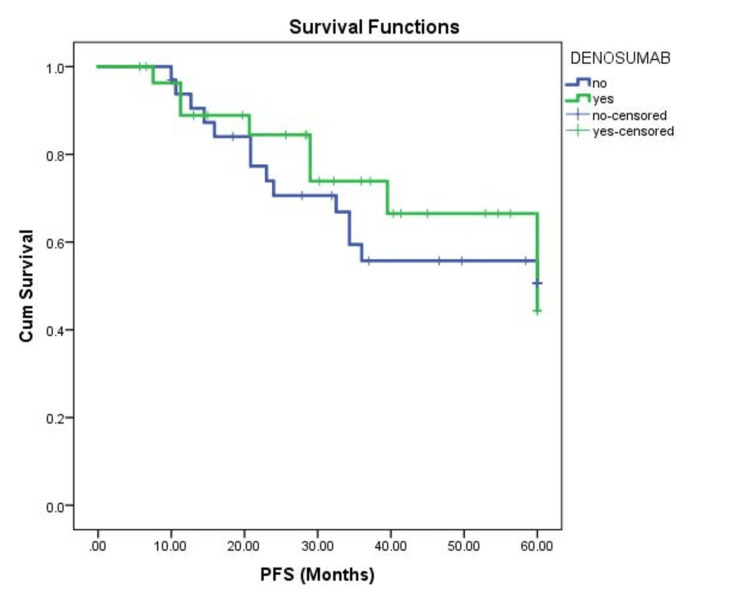
Kaplan-Meier analysis comparing the PFS of patients with GCTB who received denosumab and those who did not. PFS, progression-free survival; Cum, cumulative; GCTB, giant cell tumors of the bone

Furthermore, PS at presentation (*P *= 0.86) and the site of the tumor (*P *= 0.22) had no effect on PFS in the patient population. Although patients who received denosumab had numerically longer PFS, no statistically significant difference was noted between those treated with denosumab and those who did not (*P *= 0.603) (Figure 3). Similarly, a univariate Cox proportional hazards model found no impact of denosumab on PFS (hazard ratio [HR] = 0.634, 95% confidence interval [CI] = 0.255-1.572, *P *= 0.325).

## Discussion

GCTB is a rare, locally aggressive tumor consisting of osteoclast-like giant cells, associated with high rates of recurrence and morbidity [9]. This study assesses the efficacy of denosumab, a monoclonal antibody that targets RANKL, in the treatment of GCTB. The results of this retrospective study showed that denosumab treatment was associated with a numerical increase in PFS, but the difference was not statistically significant.

Although benign GCTB has high survival rates, the cumulative incidence of malignant GCTB is 4% [10,11]. Treatment of GCTB largely depends on the curability of the neoplasm [12]. In resectable GCTB, joint-sparing resection is the first line of treatment [12]. Preoperative denosumab, the only drug approved by the United States Food and Drug Administration for the treatment of GCTB, has recently been added to the standard GCTB treatment regimen to assist in surgical downstaging of the tumor [12]. Additionally, several studies have also highlighted the drug’s utility in unresectable GCTB [12]. However, data regarding denosumab’s effectiveness in GCTB remain limited, especially in the Middle Eastern population.

To the best of our knowledge, this study consists of the largest patient population assessing denosumab-related GCTB outcomes in the Middle Eastern literature. The characteristics of our patient population are similar to those of previous studies. For example, a study by Rockberg et al. in Sweden found that GCTB is more common in middle-aged females [13]. Another study by Verschoor et al. in the Netherlands also found similar results [14]. Contrary to similar literature, upper limb tumors were slightly more prevalent than lower limb in our patients [2,15].

The current study evaluated the effect of denosumab on PFS in patients with GCTB. PFS in our patient group is similar to that reported in the literature. Although long-term studies are limited, short-term PFS in our study is similar to other studies, which have reported one- and two-year PFS rates of 92.8% and 81%, respectively [16,17]. Similar to our results, Chawla et al. reported that median PFS was not reached in their open-label phase II trial of 532 patients with GCTB [18]. Overall, Saudi patients with GCTB have similar survival outcomes to other patients of different ethnicities.

The long-term effects of denosumab have not been studied extensively [19]. Several studies have reported decreased relapse rates in patients receiving denosumab therapy [20]. However, systematic analyses have found no association between denosumab administration and local recurrence, regardless of the administered dose [21,22]. A study by AlYami et al. in Saudi Arabia found no significant difference between patients who received denosumab and surgery versus those who only underwent surgery [2]. Similarly, our results indicate that denosumab has no effect on five-year PFS (progression, recurrence, and mortality).

The major limitation of our study is that it is a single-center study, which limits the generalizability of the study to patients across Saudi Arabia. Similarly, our study cannot represent all patients with GCTB in the Middle East due to the uni-ethnic population at our center. Furthermore, although our sample size is large relative to other studies in the area, the study remains small and may limit the power of the study and the accuracy of our results. Due to the retrospective nature of the study, there may be inherent selection biases between the two groups of patients. Finally, our study does not focus on whether recurrence is increased in denosumab patients, as this has already been assessed previously in patients in the region [2]. Nevertheless, our study provides further insight into the role of denosumab in patients with GCTB of the region. Larger long-term, multicenter studies in the region are needed to confirm our findings.

## Conclusions

Our study provides preliminary data on denosumab in Middle Eastern patients with GCTB and finds no impact of denosumab on PFS. Additionally, age, sex, BMI, PS at presentation, and tumor location had no effect on PFS survival in our population. However, due to the retrospective nature of the study and limited sample size, larger, more robust studies are needed to confirm our findings.
